# Managing Exercise-Related Glycemic Events in Type 1 Diabetes: Development and Validation of Predictive Models for a Practical Decision Support Tool

**DOI:** 10.2196/68948

**Published:** 2025-10-10

**Authors:** Sisi Ma, Ryan Coopergard, Mark Clements, Lisa Chow

**Affiliations:** 1 Department of Medicine, Medical School, University of Minnesota, Minneapolis, MN, United States; 2 Institute for Health Informatics, University of Minnesota, 420 Delaware Street SEMinneapolis, MN, 55401, United States, 1 6126267788; 3 School of Medicine, University of Missouri–Kansas City, Kansas City, MO, United States; 4 Division of Diabetes, Endocrinology and Metabolism, Department of Medicine, Medical School, University of Minnesota, Minneapolis, MN, United States

**Keywords:** type 1 diabetes, exercise-induced glycemic events, continuous glucose monitoring, predictive modeling, decision support tool, hypoglycemia, hyperglycemia

## Abstract

**Background:**

Exercise is an important aspect of diabetes self-management. Patients with type 1 diabetes frequently struggle with exercise-induced hyperglycemia and hypoglycemia, decreasing their willingness to exercise.

**Objective:**

We aim to build accurate and easy-to-deploy models to forecast exercise-induced glycemic events in real-world settings.

**Methods:**

We analyzed free-living data from the Type 1 Diabetes Exercise Initiative study, where adults with type 1 diabetes wore a continuous glucose monitor (CGM) while performing video-guided exercises (30-minute exercises at least 6 times over 4 weeks), along with concurrent detailed phenotyping of their insulin program and diet. We built models to predict glycemic events (blood glucose ≤54 mg/dL, ≤70 mg/dL, ≥200 mg/dL, and ≥250 mg/dL) during and 1 hour post exercise with variables from 4 data modalities, such as demographic and clinical (eg, glycated hemoglobin; CGM (blood glucose value and their summary statistics); carbohydrate intake and insulin administration; and exercise type, duration, and intensity. We used repeated stratified nested cross-validation for model selection and performance estimation. We evaluated the relative contribution of the 4 input data modalities for predicting glycemic events, which informs the cost and benefit for including them in the decision support tool for risk prediction. We also evaluated other important aspects related to model translation into decision support tools, including model calibration and sensitivity to noisy inputs.

**Results:**

Our models were built based on 1901 exercise episodes for 329 participants. The median age for the participants was 34 (IQR 26‐48) years. Of the participants, 74.8% (246/329) are female and 94.5% (311/329) are White. A total of 182/329 (55.3%) participants used a closed-loop insulin delivery system, while the rest used a pump without a closed-loop system. Models incorporating information from all 4 data modalities showed excellent predictive performance with cross-validated area under the receiver operating curves (AUROCs) ranging from mean 0.880 (SD 0.057) to mean 0.992 (SD 0.001) for different glycemic events. Models built with CGM data alone have statistically indistinguishable performance compared to models using all data modalities, indicating the other 3 data modalities do not add additional information with respect to predicting exercise-related glycemic events. The models based solely on CGM data also showed outstanding calibration (Brier score ≤0.08) and resilience to noisy input.

**Conclusions:**

We successfully constructed models to forecast exercise-induced glycemic events using only CGM data as input with excellent predictive performance, calibration, and robustness. In addition, these models are based on automatically captured CGM data, thus easy to deploy and maintain and incurring minimal user burden, enabling model translation into a decision support tool.

## Introduction

Exercise refers to intentional physical activity, such as aerobic, resistance, or high-intensity interval training [1]. It has many benefits, including improving overall health and longevity [2]. However, patients with type 1 diabetes (T1DM) often struggle to meet recommended exercise guidelines (≥150 minutes of moderate activity per week) [3]. One contributing factor is that exercise can cause significant glycemic variability, with strenuous exercise causing exercise-associated hyperglycemia and subsequent postexercise hypoglycemia resulting from heightened postexercise insulin sensitivity [4]. Fear of exercise-associated glycemic variability presents a significant barrier to regular physical activity in adults with T1DM [5]. On the other hand, exercise is an important aspect of diabetes self-management. Less exercise during the COVID-19 pandemic was associated with poorer glycemic control in people with T1DM and type 2 diabetes [6]. Glycemic management peri-exercise presents an ongoing challenge for patients with T1DM. To address the needs of patients, we aim to develop models and decision support tools that predict glycemic events safely and accurately with minimal user burden.

As indicated in several recent reviews and meta-analyses [7-10], many studies have explored predicting glycemic events (hypo- and hyperglycemia) using statistical and machine learning methods and demonstrated good predictive performances. However, there is evidence that a general-purpose glycemic event prediction model has reduced performance when applied to high-activity conditions [11]. A smaller set of studies focused on the prediction of glycemic event risk specifically during and post exercise [12-16] reported highly promising results. The studies using data collected from highly structured clinical studies with controlled meal intake and exercise programs achieved very strong to near-perfect predictivity for hypoglycemia (blood glucose <70 mg/dL) during and post aerobic exercise [12,13]. Studies using data collected in real-world free-living conditions reported highly promising predictivities, ranging area under the receiver operating characteristic curve (AUROC) from 0.79 to 0.84, for predicting hypoglycemia during and post exercise [15,16].

Building on prior work, we aim to address important remaining questions related to model translation into a decision support tool for exercise-related glycemic event risk prediction. Specifically, we used the T1DEXI adult dataset and constructed models to predict a larger set of glycemic events, including hypoglycemia (≤54 mg/dL and ≤70 mg/dL) and hyperglycemia (≥200 mg/dL and ≥250 mg/dL) both during and post exercise, expanding the outcome, that is, blood glucose ≤70 mg/dL during exercise, previously examined in the study by Bergford et al [16]. There are three study goals: (1) to build high-quality predictive models for exercise-induced glycemic events using data from various modalities, including patient demographics, clinical data, CGM, carbohydrate and insulin intake, and exercise characteristics; (2) to assess the contributions of the different data modalities for predicting glycemic events to inform cost-benefit trade-offs for model deployment; and (3) to refine predictive models with the aim of minimizing user input while remaining a decision support tool for effective management of glycemic events.

## Methods

### Study Cohort

We used data from the Type 1 Diabetes Exercise Initiative (T1DEXI) study [17]. The T1DEXI was a real-world study of at-home exercise in adults with T1DM, where adult participants were randomly assigned to complete 6 structured aerobic, interval, or resistance exercise sessions over 4 weeks. Each exercise was approximately 30 minutes, consisting of a 3-minute warm-up and cool-down. In addition to study exercises, participants also continued their typical forms of daily physical activity. Information regarding carbohydrate intake was collected through the T1DEXI app. Insulin dosing information was extracted from the insulin pump. Continuous glucose monitoring (CGM) data were collected using Dexcom G6 sensors (1 measurement every 5 minutes). Diabetes history, glycated hemoglobin A_1c_ (HbA_1c_), and demographics were self-reported and collected via a portal.

### Analysis Design

#### Overall Design

**Figure 1.**
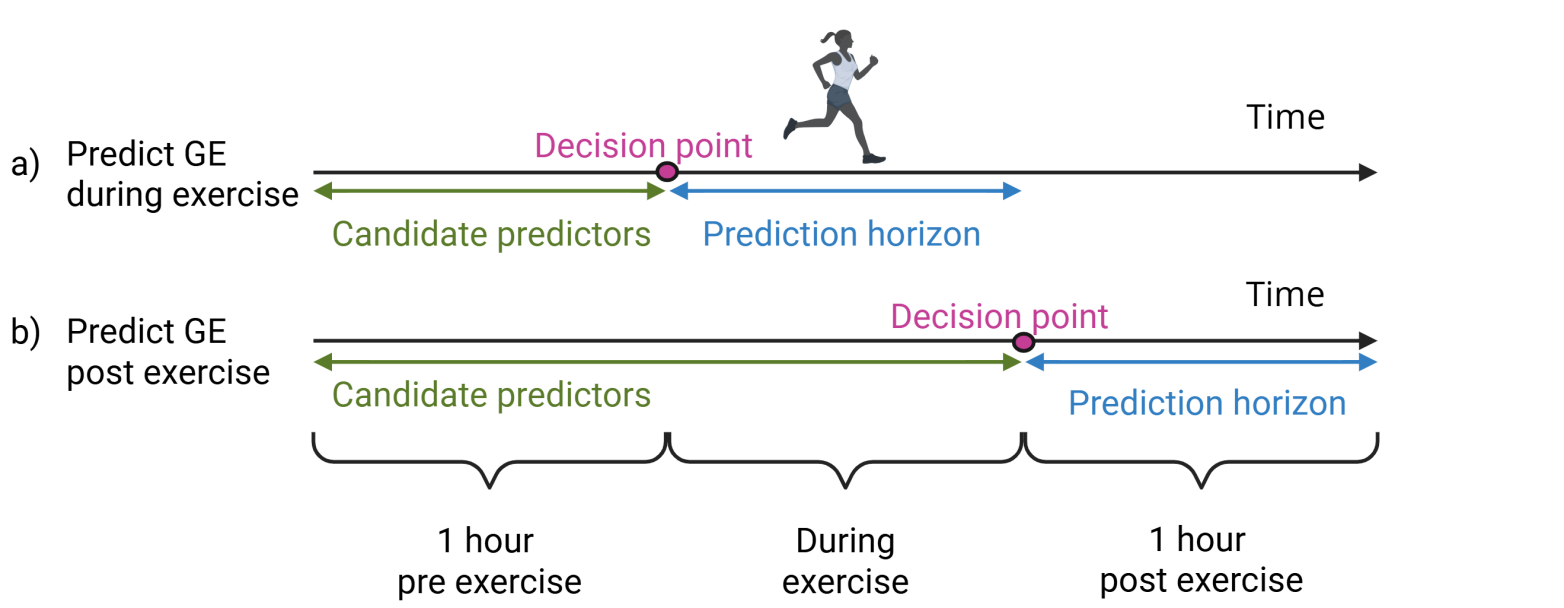
Design of analytical experiments. Subparts (A) and (B) represent the designs for predicting glycemic events during and post exercise, respectively. GE: glycemic events.

**Figure 2.**
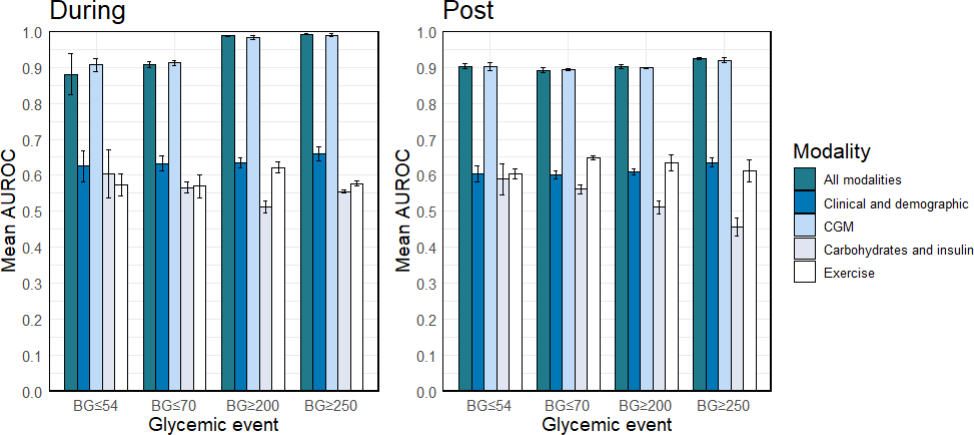
Predictive performances for the models using data from individual data modalities compared to models that use all 4 modalities for each Glycemic Event. AUROC: area under the receiver operating characteristic curve; BG: blood glucose; CGM: continuous glucose monitor.

Figure 1 illustrates the analysis design. We designed our model to assess the risk for hypoglycemic events at two critical decision points (Figure 1) for managing exercise-related glucose events for patients with T1DM (as marked by purple dots in Figure 1): (1) prior to the start of the exercise to evaluate the risk of glycemic events during exercise using data collected from 1-hour pre-exercise (Figure 1A) and (2) right after the completion of the exercise to evaluate the risk of glycemic events for the 1-hour post exercise using data collected from 1-hour pre-exercise and during exercise (Figure 1B). With glycemic event risk estimated accurately at these decision points, patients can make adjustments, for example, to their exercise plan and carbohydrate and insulin intake, to avoid glycemic events accordingly. Data from various data modalities were considered as candidate predictors, including patient demographics, clinical, CGM, carbohydrate and insulin intake, and exercise characteristics.

#### Targets of Interest

Our targets of interest are binary variables representing the occurrence of glycemic events during and 1 hour post exercise. Specifically, we examined 4 glycemic events, including severe hypoglycemia (glucose ≤54 mg/dL), hypoglycemia (glucose ≤70 mg/dL), hyperglycemia (glucose ≥200 mg/dL), and severe hyperglycemia (glucose ≥250 mg/dL). Each of the 4 glycemic events was assessed separately during the exercise and postexercise period, resulting in 8 outcomes.

#### Candidate Predictors

We extracted and constructed the following data elements as candidate predictors. All candidate predictors and their construction are listed in Table S1 in Multimedia Appendix 1.

1. Demographic data: Age, race, and sex.
2. Clinical data: Height, weight, and BMI at the beginning of the study. Most recent HbA_1c_ prior to the study period. Out of the 329 participants in our study, 318 reported an HbA_1c_ within 12 months of the start of the study.
3. CGM data: For predicting during exercise glycemic events, we used CGM data 1 hour pre-exercise (Figure 1A). For predicting postexercise glycemic events, we used CGM data 1 hour pre-exercise and during exercise. Up to 12 raw CGM readings prior to the decision point, as well as the minimum, maximum, mean, SD, and coefficient of variation of the CGM reading from the appropriate time period, were constructed and used as candidate predictors.
4. Carbohydrate and insulin intake: For predicting glycemic events during exercise, we used carbohydrate and insulin intake data 1-hour pre-exercise (Figure 1). For predicting postexercise glycemic events, we used carbohydrate and insulin intake data 1 hour pre-exercise and during exercise. We used the total amount of carbohydrates in grams from the appropriate time period and the total insulin intake, including both basal and bolus insulin, from the appropriate time period.
5. Exercise data: The study arm (aerobic, interval, or resistance exercise), the self-reported exercise intensity, and the exercise time of day were included as candidate predictors. The time of day of exercise was determined by the time of day of the start of exercise, split into the categories morning, afternoon, and night. Morning, afternoon, and night were defined as the periods of time between 5 AM and noon, between noon and 5 PM, and between 5 PM and 5 AM, respectively. For predicting postexercise glycemic events, we also included self-reported exercise duration as a candidate predictor for postexercise glycemic event prediction.

### Overview of Models and Analysis

To address the three goals mentioned above, we conducted three sets of analyses.

1. To achieve our first goal, that is, building high-quality predictive models for exercise-induced glycemic events using all available data, we built predictive models for all 8 outcomes of interest using variables from all data modalities, including patient demographics, clinical data, CGM data, carbohydrate and insulin intake, and exercise characteristics.
2. To achieve our second goal, that is, assessing the contribution of different data modalities for predicting glycemic events, we built models for all 8 outcomes of interest using data from each of the following 4 data modalities individually: patient demographics and clinical data, CGM data, carbohydrate and insulin intake, and exercise characteristics.
3. To achieve our third goal, that is, refining predictive models with the aim of clinical translation, we explored various methods to improve different aspects of model translation, including building models with variables that are easier to measure, enhancing model calibration, and assessing model performance with noisy data.

### Data Processing

To ensure sufficient data were available for each data modality to enable the comparison among data modalities (the second study goal), we included participants who had at least 80 recorded carbohydrate intakes, 6000 CGM reads, and 200 insulin intakes over the 4-week period (Figure S1 in Multimedia Appendix 1 shows the distribution of number of carbohydrate intakes, number of CGM reads, and number of insulin intakes). We did not include participants that are on multiple daily injections. Regarding exercise events, we only included study exercises (ie, the video-guided exercise as part of the TIDEXI study), since the types of exercise were well defined. To be able to compute candidate predictors, we included exercise events that had at least 10 CGM measurements 1 hour pre-exercise, 2 CGM measurements during exercise, and 10 CGM measurements 1 hour post exercise. Of the 2021 total number of exercises, 120 were filtered out with the criterion described above, resulting in 1901 exercise events. Of the 1901 exercise events, 1895 had more than 4 CGM readings. The median number of CGM readings per exercise was 6 (IQR 4-6 readings; spanning 30 minutes). The final dataset included 1901 exercise events from 329 participants.

### Methods for Predictive Modeling

#### Model Selection, Performance Estimation, and Validation

We conducted model selection and performance estimation using stratified 5-fold nested cross-validation (NCV). The inner loop of the NCV was used to select the best classifier, feature selection method, and hyperparameter combinations while the outer loop evaluated the performance of the selected models. The NCV procedures were repeated 4 times to reduce the variation associated with splitting the data into 5 folds. In our primary analysis, exercise episodes were randomly distributed into the folds of the NCV, which means that different exercise episodes from the same participant could appear in both training and testing folds. We also conducted a sensitivity analysis where multiple exercise episodes from the same individual were included in the same NCV fold to account for person-specific effects (results are similar to our primary analysis and reported in Tables S2 and S3 in Multimedia Appendix 1).

#### Feature Selection

For feature selection, we used all features, support vector machine recursive feature elimination [18], and generalized local learning, parent children (GLL-PC with the conditioning set size parameter K=1, 2, or 3) [19]. In theory, GLL-PC can select a parsimonious set of variables while retaining maximal information content regarding the prediction target. GLL-PC has also been shown to be highly successful in real-world applications [19,20].

#### Classification

We considered the following classifiers: logistic regression, random forest (with mtry=sqrt [number of variables], number of trees=500), and support vector machines with a polynomial kernel (with polynomial degree paramter *P*=1, 2, and the box constraint parameter C=0.1, 1, 10).

#### Missing Values

Treatment for missing values was incorporated into the modeling pipeline to ensure that imputation on the validation data was performed according to the distribution of the training data, preventing information leakage. Missing values for non-CGM predictor variables were handled using median imputation. Missing CGM values were replaced with the last nonmissing observation carried forward if available, or the next value carried backward otherwise. If multiple CGM values were missing consecutively, the missing value will be progressively carried forward or backward, if possible. For example, in the following sequence of measurements {t_0_, t_1_, t_2_, t_3_, t_4_, t_5_}, if t_2_, t_3_, t_4_ are missing, they will all be imputed with the value of t_1_, that is, the last nonmissing observation carried forward in time. We ensured that observations from the prediction horizon (ie, during exercise or post exercise) were never carried backward to be used as features. The percentage of missing CGM values ranged from 0% to 20% across different exercise events, with 99.5% of events having at most 10% missing CGM data.

#### Performance Metric

We used the AUROC, sensitivity, and specificity to evaluate the predictive performance of the models. All performance metrics were estimated within the NCV pipeline to obtain unbiased estimates. To estimate sensitivity and specificity in each fold of the outer loop, we first determined the thresholds in the inner loops that maximized the J-index (J=sensitivity+specificity–1). To compare the sensitivity and specificity of our model to prior literature, we applied thresholds to match the sensitivity of the previously reported models. We then calculated the sensitivity and specificity on the outer loop fold using the mean of these thresholds. We used the Brier score to evaluate model calibration.

#### Information Content Analysis

To examine the predictive performance of the 4 different data modalities in the dataset (demographics and clinical data, CGM data, carbohydrate and insulin intake, and exercise characteristics), we trained classifiers on each modality individually and compared the predictive performance to the model trained with all features.

### Methods for Improving Model Translation to Decision Support Tools

#### Models With Low Complexity for Deployment and User Burden

When translating models to decision support tools, the complexity of constructing, diagnosing, and maintaining the tool is a key consideration. Models that include variables collected from different sources entail a more complex decision support tool. This complex decision support tool is more prone to failure in real-world settings and requires more resources to maintain. In addition, variables from data sources that are not automatically captured (eg, carbohydrate intake) need to be manually inputted, resulting in increased user burden. We compare models using all 4 data sources to assess whether models based on single, easy-to-acquire data sources performed similarly to models using multiple data sources (ie, the information content analysis from the “Information Content Analysis” section).

#### Model Performance With Noisy Data

To assess the robustness of our models to noise in CGM data at prediction time, we added Gaussian noise of different levels to the outer loop testing data and evaluated the predictive performance. This approach mimics real-world conditions where CGM measurements may contain more noise than the data used for training the model. An issue with potential noise in CGM measurements has been documented. A recent study by Skroce et al [21] demonstrated that the average CGM noise is up to 15% mean absolute relative difference across all exercise intensities investigated when compared to capillary glucose. We considered 4 different noise levels, where Gaussian noise with a mean of x and SD of p was added to each CGM measurement, with x being the original CGM value and p representing noise levels of (5%, 10%, 15%, and 20%). This corresponds to mean absolute relative differences of 4%, 8%, 12%, and 16%, respectively.

#### Model Calibration

The close correspondence between model-predicted risk and observed risk is crucial for deploying the model in a real-world setting [22]. Deviations of predicted risk from the actual risk can lead to model misinterpretation and misuse. Therefore, we evaluated the calibration of our models using the Brier Score [23]. To potentially improve model calibration, we applied isotonic regression, Platt scaling, and spline calibration [24] to recalibrate the model predictions.

#### Analysis Tools

Analyses were conducted using custom scripts in MATLAB 2023b (MathWorks) and R version 3.5.0 (R Foundation for Statistical Computing).

### Ethical Considerations

This study is based on deidentified, publicly available data and is not human subjects research.

## Results

### Participant and Exercise Characteristics

Table 1 shows characteristics of participants. The median age for the 329 participants was 34 (IQR 26‐48) years. The majority of participants were female (246/329, 74.8%) and White (331/329, 94.5%). The median HbA_1c_ was 6.5% (IQR 6.1%‐7.0%). The median BMI was 24.8 (IQR 22.86‐27.46). A total of 326 (>99%) patients were on fast- or rapid-acting insulin, while the remaining 3/329 (<1%) patients were on short-acting insulin. Notably, 18/329 (5.5%) participants were additionally taking metformin. A total of 18,329 (55.3%) participants used a closed-loop insulin delivery system, while the remaining 147/329 (44.7%) participants used a pump without a closed-loop system. The median duration of T1DM was 16 (IQR 10.75‐24) years. The median total daily insulin dose was 37.3 (IQR 28.06‐47.96) units. There were similar numbers of participants in each exercise intervention group: 122/329 (34%) in the aerobic training group, 113/329 (34.3%) in the interval training group, and 104/329 (31.6%) in the resistance training group. Over the 4 weeks of the study, the median number of exercise events per participant was 6 (IQR 4‐6).

**Table 1.** Participant characteristics reported as median and IQR for continuous variables, and count and percentage for discrete variables.

Variable name		Descriptive statistics
Age (years), median (IQR)		34 (26-48)
Sex, n (%)		
Male		83 (25.2)
Female		246 (74.8)
Race, n (%)		
White		311 (94.5)
Black		1 (0.3)
Asian		5 (1.5)
American Indian or Alaska Native		1 (0.3)
Other		5 (1.5)
Number of medications, median (IQR)		2 (2-2)
Insulin delivery method, n (%)		
Closed-loop		182 (55.3)
Pump without closed-loop		147 (44.7)
Hemoglobin A_1c_ (%), mean (SD)		6.56 (0.66)
Number of participants per exercise type, n (%)		
Aerobic		122 (34)
Resistance		104 (31.6)
Interval		113 (34.3)
Number of exercises per participant, median (IQR)		6 (5-6)
Number of peri-exercise glycemic events per participant, median (IQR)		
≤54 during exercise		0 (0-0)
≤70 during exercise		0 (0-0)
≥200 during exercise		1 (0-1)
≥250 during exercise		0 (0-0)
≤54 post exercise		0 (0-0)
≤70 post exercise		0 (0-1)
≥200 post exercise		1 (0-1)
≥250 post exercise		0 (0-0)

### Characteristics of Exercise Events

Table 2 summarizes the characteristics of exercise events. Of the 1901 exercise events included in the analysis, the median duration was 30 (IQR 22‐30) minutes, with on average 12.4% (SD 14.7%) of missing CGM measurements during the exercise, ranging from 0% to 66.7%. Similar numbers of exercise episodes were observed for each exercise type, with 649 aerobic exercises, 659 interval exercises, and 593 resistance exercises. A total of 596 (27.8%) of the 1901 exercises started in the morning. Severe hypoglycemia during and post exercise was observed, and morning exercise was less frequent. Only 0.17% of morning exercises resulted in hypoglycemic events (≤54 mg/dL) during exercise, compared to 1.23% observed during exercises at other times of the day (*χ*^2^_1_=4.1; *P*=.04). Severe hypoglycemia (≤54 mg/dL) post exercise occurred in 1.84% of morning exercises, compared to 4.75% at other times of the day (*χ*^2^_1_=8.5; *P*=.003). There was no statistically significant difference in the likelihood of hypoglycemic events (≤54 mg/dL) across different exercise types (*χ*^2^_2_=2.1; *P*=.35).

**Table 2.** Exercise characteristics reported as median and IQR for continuous variables, and count and percentage for discrete variables.

Variable names		During exercise				Post exercise			
		Hypoglycemia		Hyperglycemia		Hypoglycemia		Hyperglycemia	
	Total	≤54	≤70	≥200	≥250	≤54	≤70	≥200	≥250
Number of exercise events, n (%)	1901	17 (0.9)	88 (4.6)	286 (15)	76 (4)	73 (3.8)	255 (13.4)	315 (16.6)	92 (4.8)
Exercise duration (minutes), median (IQR)	30 (22-30)	30, (30-30)	30 (24.5-30)	30 (22-30)	30 (22-30)	30 (22-30)	28 (22-30)	30 (22-30)	30 (24.5-30)
Number of morning exercises, n (%)	529	1 (0.2)	15 (2.8)	77 (14.6)	14 (2.6)	11 (2.1)	60 (11.3)	92 (17.4)	25 (4.7)
Number of evening exercises, n (%)	780	10 (1.3)	48 (6.2)	129 (16.5)	36 (4.6)	41 (5.3)	127 (16.3)	136 (17.4)	40 (5.1)
Number of aerobic exercises, n (%)	649	3 (0.5)	30 (4.6)	102 (15.7)	22 (3.4)	27 (4.2)	97 (14.9)	104 (16)	35 (5.4)
Number of interval exercises, n (%)	659	7 (1.1)	32 (4.9)	91 (13.8)	31 (4.7)	28 (4.2)	92 (14.0)	111 (16.8)	27 (4.1)
Number of resistance exercises, n (%)	593	7 (1.2)	26 (4.4)	93 (15.7)	23 (3.9)	18 (3)	66 (11.1)	100 (16.9)	30 (5.1)

### Models Forecasting Exercise-Related Glycemic Events

We built a set of models using variables from all available data modalities, including patient demographics and clinical data, exercise characteristics, CGM data, and insulin and carbohydrate intake data. The forecasting performance for all glycemic events during and after exercise was excellent, with mean AUROC values ranging from 0.880 to 0.992 (Table 3). The AUROCs for predicting glycemic events during exercise were generally higher compared to those for postexercise prediction. It is worth noting that, although the modeling process had access to all 28 variables (for during-exercise predictions) or all 35 variables (for postexercise predictions) from the 4 data modalities (patient demographics, clinical data, CGM data, and carbohydrate and insulin intake), feature selection was performed to optimize predictive performance. This process resulted in models that used only a subset of the available variables, as shown in Table 3. The number of variables selected in each model varied from 2 to 35. We included model coefficients or variable importance of all models in Table 3 and Table S4 in Multimedia Appendix 1.

**Table 3.** Predictive performances of models using data from all four modalities for each glycemic event at different prediction horizons (measured by area under the receiver operating characteristic curve) and number of predictors from each data module that was selected into the model by feature selection.

Glycemic event			AUROC^b^		Number of predictors				
			Mean (SD)		Total	CGM^a^	Clinical and demographics	Exercise	Carbohydrate and insulin
During									
Hypo									
≤54			0.880 (0.057)		28	17	7	2	2
≤70			0.907 (0.009)		8	7	0	1	0
Hyper									
≥200			0.987 (0.001)		2	2	0	0	0
≥250			0.992 (0.001)		2	2	0	0	0
Post									
Hypo									
≤54			0.902 (0.007)		8	7	1	0	0
≤70			0.892 (0.006)		14	14	0	0	0
Hyper									
≥200			0.901 (0.006)		35	22	7	2	4
≥250			0.924 (0.003)		13	11	1	0	1

a CGM: continuous glucose monitoring.

b AUROC: area under the receiver operating characteristic curve.

### Models Built Using Only CGM Data

To determine the predictive capability of individual data modalities, we developed models using each data modality separately. Figure 2 summarizes the performance of these models. The left panel shows glycemic events during exercise, and the right panel represents glycemic event post exercise. The height and error bar of the bar plots represent the mean and SD of AUROC. As shown in Figure 2 and Table S2 in Multimedia Appendix 1, the models constructed with the CGM data resulted in excellent AUROC ranging from 0.894 to 0.989. The predictive performance of these models was not statistically significantly different from models using all data modalities for any glycemic event outcomes (*P*>.05). Other data modalities showed low predictive power (mean AUROCs ≤0.659), with significantly lower performance compared to the full model (*P*<.01). When considered collectively, these results indicate that incorporating patients’ demographic traits, clinical condition, carbohydrate and insulin consumption, and exercise variables, such as timing and type, fails to furnish additional information regarding exercise-induced glycemic events beyond what is provided by the CGM data.

We included the CGM-only model parameters or variable importance in Table S5 in Multimedia Appendix 1, and we compared the performance of the CGM-only models with models trained on a single CGM measurement in Table S8 in Multimedia Appendix 1. We also included an executable version of these models.

### Deployment Advantages of Models Using Only CGM Features

#### Deployment Complexity and User Burden

The CGM-only model is preferable for deployment due to its high predictive performance and lower complexity of deployment. As stated previously, for all 8 glycemic outcomes, the model with all data modalities did not perform statistically significantly better compared to the CGM-only model, indicating the glycemic outcomes can be predicted equally well with and without the other variables, such as demographics, clinical variables, carbohydrate and insulin, and exercise characteristics. This is advantageous for model deployment. Since CGM data is automatically captured, it can be easily fed into the prediction model without user intervention, reducing the burden on patients. In contrast, real-world carbohydrate data is difficult to measure accurately, and collecting this data incurs an additional burden on patients. Data from insulin pumps are automatically documented, but extracting and integrating insulin pump data also adds additional complexity to a decision support tool for forecasting glycemic events.

#### Noise Tolerance

**Figure 3.**
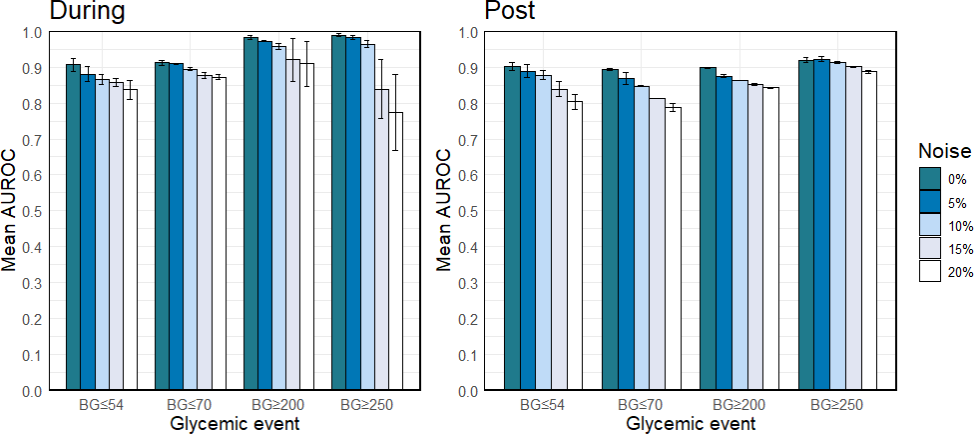
Predictive Performance (area under the receiver operating characteristic curve) of Model using only continuous glucose monitoring features with different amounts of added noise in the continuous glucose monitoring features data. AUROC: area under the receiver operating characteristic curve; BG: blood glucose.

Another consideration of model deployment is the impact of measurement noise in CGM data. Different individuals’ CGM measurements often exhibit varying levels of noise, influenced by factors such as sensor calibration, activity level, and pressure on the sensor [21,25-28]. We empirically assessed the performance degradation of our models under different levels of noise (5%, 10%, 15%, and 20%) to the CGM data at prediction time (Figure 3 and Table S3 in Multimedia Appendix 1). In Figure 3, the left panel shows glycemic events during exercise, and the right panel represents glycemic events post exercise. The height and error bar of the bar plots represent the mean and SD of the AUROC. As expected, increased noise led to a reduction in model performance. However, the degradation was generally moderate, except for predicting hyperglycemia (blood glucose ≥250 mg/dL) during exercise, where a decrease in AUROC of 0.216 was observed with 20% noise. For the other 7 out of the 8 glycemic events across different prediction horizons, the decreases in AUROC were small to moderate (<0.12) even when up to 20% noise was added.

We also examined whether models built with additional data modalities (beyond CGM) demonstrated greater resilience to added noise (Table S3 in Multimedia Appendix 1). We found no statistically significant difference in the AUROCs between the CGM-only models and those incorporating additional data modalities. This suggests that the CGM-only model has a similar level of noise tolerance compared to the more complex models using multiple data sources, further supporting the use of the CGM-only model in real-world settings due to its simplicity and robustness.

#### Sensitivity, Specificity, and Calibration

Model sensitivity, specificity, and calibration are crucial factors for determining how and when a model should be applied in real-world settings. Table 4 presents the sensitivity, specificity, and calibration results of the CGM-only model. Sensitivity and specificity are influenced by the threshold used to binarize the continuous model predictions. We report the mean and SD from the NCV outer loop for sensitivity and specificity using a threshold that maximized the J-index in the inner loop of the NCV. Generally, our models showed good sensitivity (>0.76, except for hypoglycemia ≤54 mg/dL) and high specificity (>0.80 for all outcomes). In comparison with other studies, our models demonstrated similar or better sensitivity at equivalent specificity levels (Table S6 in Multimedia Appendix 1).

**Table 4.** Sensitivity, specificity, and calibration results of the continuous glucose monitoring features–only models.

Event type			Sensitivity, mean (SD)	Specificity, mean (SD)	Brier score, mean (SD)	
					Without recalibration	With recalibration
During						
Hypo						
≤54			0.667 (0.193)	0.893 (0.065)	0.017 (0.008)	0.006 (0.003)
≤70			0.770 (0.014)	0.909 (0.011)	0.035 (0.009)	0.034 (0.010)
Hyper		
≥200			0.931 (0.005)	0.943 (0.039)	0.040 (0.034)	0.029 (0.006)
≥250			0.905 (0.022)	0.978 (0.003)	0.010 (0.000)	0.011 (0.004)
Post						
Hypo						
≤54			0.799 (0.047)	0.802 (0.016)	0.033 (0.005)	0.033 (0.011)
≤70			0.787 (0.018)	0.825 (0.012)	0.077 (0.001)	0.072 (0.014)
Hyper						
≥200			0.762 (0.019)	0.867 (0.014)	0.075 (0.001)	0.080 (0.009)
≥250			0.790 (0.010)	0.899 (0.024)	0.028 (0.001)	0.023 (0.006)

We also evaluated the Brier score for model calibration before and after applying different calibration methods (Table 4). The models achieved excellent calibration performance without additional calibration methods (Brier score <0.1 for all models). Applying calibration techniques did not significantly improve calibration for any of the glycemic outcomes (adjusted *P*>.05). Table 4 shows the Brier scores before and after applying the isometric regression. Results using Platt and spline calibration methods are similar and reported in Table S7 in Multimedia Appendix 1.

## Discussion

### Principal Findings

This study has made several key contributions. First, we leveraged a large and comprehensive dataset capturing free-living exercise episodes of participants with T1DM and built high-quality predictive models for exercise-induced glycemic events. These models used data from multiple modalities, including patient demographics, clinical data, CGM data, carbohydrate and insulin intake, and exercise characteristics. The predictive performance of our models for all examined exercise-induced glycemic events was excellent, with mean AUROCs >0.880 (SD 0.057). Second, we empirically assessed the information content of individual data modalities. Our results showed that the predictive performance of models using only CGM data was statistically indistinguishable from models incorporating variables from all data modalities. This suggests that a high-quality and cost-effective decision support tool can be built solely based on CGM data, resulting in cost savings. Third, we demonstrated the CGM-based models’ good calibration and robustness to noisy inputs, in addition to excellent predictive performance. Our CGM-based models have strong potential to be translated into a decision support tool that is easy to deploy and maintain, offering support for patients with T1DM.

### Comparison to Prior Work

This study is among the few that focus on predicting exercise-induced glycemic events for the adult T1DM population in nonlaboratory settings using a large sample size. Our models demonstrated similar performances for exercise-related glycemic events compared to previously reported studies [12-16] (Table S4 in Multimedia Appendix 1). We made additional contributions by building models for a larger variety of outcomes.

### Strength

A major strength of this study is that we assessed and refined the models for translating into decision support tools that are accurate, robust, and easy to deploy and maintain, while minimizing the burden on patients. To the best of our knowledge, this is the first study to examine multiple crucial aspects of model translation into decision support tools for managing exercise-related glycemic events in T1DM.

### Limitations

The first limitation relates to the dataset. We choose to derive our models based on the T1DEXI, since it is one of the largest and most comprehensive datasets available that studies glucose control peri-exercise in the real-world setting. Despite its comprehensiveness, the T1DEXI population is relatively young (median age 34, range 18-69, IQR 26-48 years) years and predominantly White, which may limit the generalizability of the models to the broader clinical T1DM population. Similarly, the exercise episodes in the T1DEXI were relatively uniform in duration and self-reported intensity, which may not reflect the full diversity of real-world exercise activities. The second limitation relates to the types of variables examined related to participants and exercise episodes. Although our findings suggest that CGM data alone contains similar predictive information compared to all 4 data modalities examined, including additional information, such as sleep data, more detailed dietary information, and physical activity data from accelerometers could enhance model performance and robustness. These data elements were either not available in T1DEXI or difficult to construct from available data due to lack of standardized methods. Third, while our models successfully flag exercise episodes that are more likely to result in glycemic events, they do not currently suggest specific interventions to mitigate these risks.

### Future Directions

To address the first 2 limitations mentioned previously, future work can validate models developed in this study and develop new models de novo based on data that is more comprehensive and from a more diverse population. To address the third limitation, computational causal modeling techniques [29] can be applied to a dataset containing a wide range of potentially modifiable causal factors for glycemic events. This approach can uncover personalized intervention strategies to reduce glycemic events both in the acute period during and after exercise, as well as over a longer time horizon. Further, we empirically demonstrated that models using CGM data only have statistically indistinguishable performance compared to models using data from all modalities (Figure 2, Table S2 in Multimedia Appendix 1). This phenomenon is related to the Rashomon effect and target information equivalence, that is, there are often a multitude of models with optimal and near-optimal performance [30,31]. Future work can dissect and catalog the information-equivalent variables for glycemic events. This can potentially lead to both improved mechanistic understanding of glycemic events and their care [30,32-34].

### Conclusion

We developed multiple predictive models for exercise-induced glycemic events, achieving excellent predictive performance. Notably, the models using automatically captured CGM data exhibited strong performance, are well-calibrated, are cost-effective, and are robust to noisy input data. These models can be translated into a high-performance and easy-to-deploy decision support tool. This study marks an important first step toward creating a practical decision support tool for managing exercise-induced glycemic events in patients with T1DM.

## Supplementary material

10.2196/68948 Multimedia Appendix 1 Visualizations of data availability and results from additional experiments.

## Abbreviations

AUROC: area under the receiver operating characteristic curve
CGM: continuous glucose monitoring
GLL-PC: generalized local learning, parent children
HbA_1c_: glycated hemoglobin A_1c_
NCV: nested cross-validation
T1DEXI: Type 1 Diabetes Exercise Initiative
T1DM: type 1 diabetes mellitus
