# Supplementary material for: Managing Exercise-Related Glycemic Events in Type 1 Diabetes: Development and Validation of Predictive Models for a Practical Decision Support Tool
Source: JMIR Diabetes. 2025 Oct 10;10:e68948. doi: 10.2196/68948 (PMC12513686; doi:10.2196/68948)
Supplement: Multimedia Appendix 1 [file diabetes-v10-e68948-s001.docx]

**Figure S1. The distribution of number of carb intakes, number of CGM reads, and number of insulin intakes for all participants. Participants who have at least 80 recorded carb intakes, 6000 CGM reads, and 200 insulin intakes (marked with dashed line in the corresponding subplots) over the 4 week period were included in the subsequent analyses.**


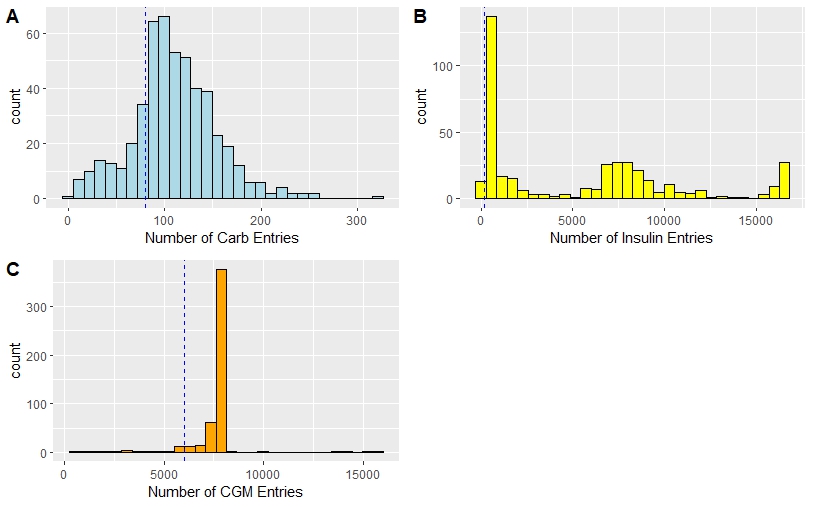


**Table S1. Descriptions for all candidate predictors. Two time windows are considered:** (1) up to 1 hour pre-exercise (2) during exercise. For predicting glycemic events during exercise, we use CGM, carbohydrate, and insulin data from time window (1). For predicting post-exercise glycemic events, we use CGM, carbohydrate, and insulin data from time window (1) and (2).

| **Modality** | **Variable** | **Variable Description/Construction** |
| --- | --- | --- |
| CGM | CGM read t minutes prior to exercise start, t= {5, 10, 15, …, 60} | We use all CGM measurements 60 min prior to the start of exercise as predictors. |
| CGM | Minimum of CGM values in time window | The minimum CGM value recorded in the specified time window |
| CGM | Maximum of CGM values in time window | The maximum CGM value recorded in the specified time window |
| CGM | Mean of CGM values in time window | The mean of the non-missing CGM values in the specified time window |
| CGM | Standard deviation of CGM values in time window | The standard deviation of the non-missing CGM values in the specified time window |
| CGM | Coefficient of variation of CGM values in time window | The coefficient of variation (i.e. standard deviation divided by the mean) of the non-missing CGM values in the specified time window |
| Carbs/Insulin | Total carbs in time window | The total grams of carbs consumed in the specified time window |
| Carbs/Insulin | Total insulin in time window | The total insulin (bolus and basal) delivered in the specified time window |
| Demo/Clinical | Race | Race of the participant (white, black/african american, asian, american indian/alaska native, multiracial, or unknown) |
| Demo/Clinical | Sex | Sex of the participant |
| Demo/Clinical | Age | Age in years |
| Demo/Clinical | A1C | Most recent hemoglobin A1C (%) prior to the study period. 318 (96.7%) reported an A1C within 12 months of study start. |
| Demo/Clinical | Height | Height of the participant in inches at the start of the study |
| Demo/Clinical | Weight | Weight of the participant in pounds at the start of the study |
| Demo/Clinical | BMI | BMI of the participant at the start of the study |
| Exercise | Time of day | The time of day at the start of exercise, categorized as morning (5am - 12pm), afternoon (12pm - 5pm), and night (5pm - 5am). |
| Exercise | Study arm | The exercise type assigned in the study: aerobic, interval, or resistance. |
| Exercise | Exercise intensity | Self-reported exercise intensity, on a scale from 0 (low intensity) to 2 (high intensity) |
| Exercise | Exercise duration (post-exercise prediction only) | Self-reported exercise duration, in minutes. |

**Table S2. Performance of models (AUROCs) built with different data modalities for different glycemic events. The ungrouped estimates represent nested cross validation AUROC estimates when exercise episodes are randomly assigned into different cross validation folds. This is the primary analysis reported in the main text of the paper and Figure 2. The grouped estimates represent nested cross validation AUROC estimates when exercise episodes from the same participant are grouped together and randomly assigned into different cross validation folds. There is no statistically significant difference in the AUROCs between these models and the models built with only the CGM data.**

|  |  | |  | **All Modalities** | | **CGM** | | **Clinical & Demographics** | | **Exercise** | | **Carbohydrates & Insulin** | |
| --- | --- | --- | --- | --- | --- | --- | --- | --- | --- | --- | --- | --- | --- |
| **Estimates** | **Event Type** | | | **mean** | **sd** | **mean** | **sd** | **mean** | **sd** | **mean** | **sd** | **mean** | **sd** |
| **Ungrouped** | **During** | **Hypo** | **≤ 54** | 0.88 | 0.057 | 0.906 | 0.017 | 0.625 | 0.042 | 0.573 | 0.03 | 0.604 | 0.066 |
|  |  |  | **≤ 70** | 0.907 | 0.009 | 0.912 | 0.007 | 0.633 | 0.022 | 0.57 | 0.032 | 0.566 | 0.015 |
|  |  | **Hyper** | **≥ 200** | 0.987 | 0.001 | 0.983 | 0.006 | 0.634 | 0.014 | 0.622 | 0.016 | 0.512 | 0.016 |
|  |  |  | **≥ 250** | 0.992 | 0.001 | 0.989 | 0.005 | 0.659 | 0.02 | 0.577 | 0.007 | 0.555 | 0.005 |
|  | **Post** | **Hypo** | **≤ 54** | 0.902 | 0.007 | 0.901 | 0.011 | 0.605 | 0.022 | 0.605 | 0.014 | 0.589 | 0.042 |
|  |  |  | **≤ 70** | 0.892 | 0.006 | 0.894 | 0.003 | 0.601 | 0.011 | 0.649 | 0.006 | 0.561 | 0.013 |
|  |  | **Hyper** | **≥ 200** | 0.901 | 0.006 | 0.898 | 0.002 | 0.61 | 0.008 | 0.634 | 0.023 | 0.511 | 0.018 |
|  |  |  | **≥ 250** | 0.924 | 0.003 | 0.919 | 0.007 | 0.636 | 0.013 | 0.612 | 0.031 | 0.457 | 0.026 |
| **Grouped** | **During** | **Hypo** | **≤ 54** | 0.92 | 0.031 | 0.899 | 0.009 | 0.478 | 0.038 | 0.588 | 0.032 | 0.56 | 0.107 |
|  |  |  | **≤ 70** | 0.911 | 0.003 | 0.907 | 0.007 | 0.531 | 0.012 | 0.56 | 0.012 | 0.62 | 0.022 |
|  |  | **Hyper** | **≥ 200** | 0.985 | 0.003 | 0.985 | 0.004 | 0.598 | 0.014 | 0.518 | 0.007 | 0.613 | 0.015 |
|  |  |  | **≥ 250** | 0.988 | 0.004 | 0.993 | 0 | 0.585 | 0.028 | 0.549 | 0.014 | 0.632 | 0.032 |
|  | **Post** | **Hypo** | **≤ 54** | 0.902 | 0.006 | 0.898 | 0.015 | 0.511 | 0.025 | 0.581 | 0.019 | 0.553 | 0.036 |
|  |  |  | **≤ 70** | 0.891 | 0.005 | 0.893 | 0.002 | 0.51 | 0.019 | 0.549 | 0.012 | 0.605 | 0.015 |
|  |  | **Hyper** | **≥ 200** | 0.896 | 0.005 | 0.895 | 0.004 | 0.606 | 0.007 | 0.497 | 0.016 | 0.597 | 0.022 |
|  |  |  | **≥ 250** | 0.928 | 0.005 | 0.927 | 0.003 | 0.588 | 0.009 | 0.501 | 0.033 | 0.597 | 0.017 |

Table S3. Performance of models built with CGM only and all 4 data modalities with different amount of noise added, for different glycemic outcomes. The ungrouped estimates represent nested cross validation AUROC estimates when exercise episodes are randomly assigned into different cross validation folds. This is the primary analysis reported in the main text of the paper and Figure 3. The grouped estimates represent nested cross validation AUROC estimates when exercise episodes from the same participant are grouped together and randomly assigned into different cross validation folds. There is no statistically significant difference in the AUROCs between these models and the models built with only the CGM data.

|  |  | **Ungrouped** | | | | | | | | **Grouped** | | | | | | | |
| --- | --- | --- | --- | --- | --- | --- | --- | --- | --- | --- | --- | --- | --- | --- | --- | --- | --- |
|  |  | **CGM-only** | | | | **All Data Modalities** | | | | **CGM-only** | | | | **All Data Modalities** | | | |
| **Glycemic Event Type** | **Percent Added Noise** | **During** | | **Post** | | **During** | | **Post** | | **During** | | **Post** | | **During** | | **Post** | |
|  |  | **AUROC** | | **AUROC** | | **AUROC** | | **AUROC** | | **AUROC** | | **AUROC** | | **AUROC** | | **AUROC** | |
|  |  | **mean** | **sd** | **mean** | **sd** | **mean** | **sd** | **mean** | **sd** | **mean** | **sd** | **mean** | **sd** | **mean** | **sd** | **mean** | **sd** |
| ≤ 54 | 0 | 0.906 | 0.017 | 0.901 | 0.011 | 0.911 | 0.015 | 0.902 | 0.007 | 0.899 | 0.009 | 0.898 | 0.015 | 0.92 | 0.031 | 0.902 | 0.006 |
| ≤ 54 | 5 | 0.88 | 0.021 | 0.889 | 0.017 | 0.885 | 0.046 | 0.888 | 0.008 | 0.882 | 0.039 | 0.886 | 0.009 | 0.869 | 0.034 | 0.879 | 0.007 |
| ≤ 54 | 10 | 0.866 | 0.013 | 0.878 | 0.012 | 0.84 | 0.056 | 0.875 | 0.008 | 0.838 | 0.036 | 0.874 | 0.008 | 0.842 | 0.031 | 0.854 | 0.019 |
| ≤ 54 | 15 | 0.856 | 0.011 | 0.838 | 0.021 | 0.844 | 0.031 | 0.841 | 0.003 | 0.823 | 0.025 | 0.844 | 0.009 | 0.821 | 0.023 | 0.816 | 0.013 |
| ≤ 54 | 20 | 0.837 | 0.027 | 0.804 | 0.021 | 0.771 | 0.098 | 0.799 | 0.012 | 0.786 | 0.047 | 0.814 | 0.032 | 0.764 | 0.059 | 0.784 | 0.009 |
| ≤ 70 | 0 | 0.912 | 0.007 | 0.894 | 0.003 | 0.907 | 0.009 | 0.892 | 0.006 | 0.907 | 0.007 | 0.893 | 0.002 | 0.911 | 0.003 | 0.891 | 0.005 |
| ≤ 70 | 5 | 0.909 | 0.002 | 0.869 | 0.016 | 0.901 | 0.007 | 0.874 | 0.007 | 0.908 | 0.006 | 0.875 | 0.003 | 0.901 | 0.01 | 0.871 | 0.001 |
| ≤ 70 | 10 | 0.894 | 0.004 | 0.847 | 0.001 | 0.89 | 0.007 | 0.846 | 0.002 | 0.895 | 0.007 | 0.848 | 0.004 | 0.893 | 0.006 | 0.845 | 0.006 |
| ≤ 70 | 15 | 0.876 | 0.009 | 0.813 | 0.001 | 0.871 | 0.01 | 0.805 | 0.005 | 0.882 | 0.001 | 0.81 | 0.005 | 0.874 | 0.006 | 0.799 | 0.007 |
| ≤ 70 | 20 | 0.872 | 0.007 | 0.788 | 0.011 | 0.861 | 0.006 | 0.781 | 0.014 | 0.868 | 0.014 | 0.79 | 0.007 | 0.86 | 0.023 | 0.783 | 0.013 |
| ≥ 200 | 0 | 0.983 | 0.006 | 0.898 | 0.002 | 0.987 | 0.001 | 0.901 | 0.006 | 0.985 | 0.004 | 0.895 | 0.004 | 0.985 | 0.003 | 0.896 | 0.005 |
| ≥ 200 | 5 | 0.972 | 0.002 | 0.875 | 0.004 | 0.971 | 0.003 | 0.88 | 0.002 | 0.97 | 0.005 | 0.872 | 0.002 | 0.97 | 0.005 | 0.87 | 0.003 |
| ≥ 200 | 10 | 0.958 | 0.009 | 0.863 | 0.001 | 0.961 | 0.002 | 0.866 | 0.003 | 0.951 | 0.024 | 0.859 | 0.005 | 0.955 | 0.014 | 0.859 | 0.004 |
| ≥ 200 | 15 | 0.921 | 0.06 | 0.851 | 0.003 | 0.951 | 0.001 | 0.855 | 0.001 | 0.925 | 0.05 | 0.85 | 0.004 | 0.928 | 0.045 | 0.846 | 0.003 |
| ≥ 200 | 20 | 0.909 | 0.062 | 0.842 | 0.001 | 0.94 | 0.002 | 0.845 | 0.004 | 0.86 | 0.157 | 0.841 | 0.003 | 0.88 | 0.117 | 0.839 | 0.005 |
| ≥ 250 | 0 | 0.989 | 0.005 | 0.919 | 0.007 | 0.992 | 0.001 | 0.924 | 0.003 | 0.993 | 0 | 0.927 | 0.003 | 0.988 | 0.004 | 0.928 | 0.005 |
| ≥ 250 | 5 | 0.982 | 0.005 | 0.922 | 0.007 | 0.937 | 0.096 | 0.919 | 0.005 | 0.98 | 0.006 | 0.924 | 0.002 | 0.917 | 0.084 | 0.922 | 0.006 |
| ≥ 250 | 10 | 0.964 | 0.009 | 0.913 | 0.003 | 0.971 | 0.008 | 0.911 | 0.005 | 0.912 | 0.127 | 0.911 | 0.004 | 0.924 | 0.102 | 0.91 | 0.008 |
| ≥ 250 | 15 | 0.838 | 0.082 | 0.901 | 0.002 | 0.876 | 0.089 | 0.903 | 0.004 | 0.887 | 0.145 | 0.904 | 0.003 | 0.899 | 0.064 | 0.901 | 0.014 |
| ≥ 250 | 20 | 0.773 | 0.106 | 0.887 | 0.004 | 0.826 | 0.138 | 0.892 | 0.002 | 0.834 | 0.129 | 0.891 | 0.003 | 0.867 | 0.046 | 0.888 | 0.014 |

**Table S4: Model coefficients or variable importance for models built with all 4 data modalities**

|  | During | | | | Post | | | |
| --- | --- | --- | --- | --- | --- | --- | --- | --- |
|  | hypo <=54 | hypo <=70 | hyper >= 200 | hyper >= 250 | hypo <=54 | hypo <=70 | hyper >= 200 | hyper >= 250 |
| Classifier | svm | glm | glm | svm, quadratic kernel* | glm | svm | glm | glm |
| Exercise Duration |  |  |  |  |  |  | 0.01 |  |
| Exercise Intensity | 0.05 |  |  |  |  |  | -0.27 |  |
| AGE | -0.03 |  |  |  |  |  | -0.02 | -0.02 |
| SD CGM before | 0.05 |  |  |  |  |  | 0.03 |  |
| Min CGM before | -0.04 |  |  |  |  |  | -0.01 |  |
| Max CGM before | -0.09 | -0.09 |  |  |  |  | -0.02 |  |
| Mean CGM before | 0.10 |  |  |  |  |  | -0.26 |  |
| CoV CGM before | 0.01 | 16.64 |  |  |  |  | -0.01 |  |
| Carbs before | -0.01 |  |  |  |  |  | 0.00 |  |
| Carbs during |  |  |  |  |  |  | 0.07 |  |
| Insulin before | -0.14 |  |  |  |  |  | -0.03 |  |
| Insulin during |  |  |  |  |  |  | -0.02 | 0.15 |
| CGM 5 min before | -0.25 | -0.22 | 0.23 | 0.29 | 0.11 | 0.02 | 0.01 | -0.12 |
| CGM 10 min before | 0.18 | 0.12 |  | 0.16 | -0.02 | 0.03 | 0.01 | 0.08 |
| CGM 15 min before | 0.00 | 0.11 | -0.10 |  |  | -0.02 | 0.01 | 0.01 |
| CGM 20 min before | 0.12 | -0.02 |  |  |  | -0.01 | 0.00 | -0.10 |
| CGM 25 min before | -0.15 |  |  |  |  | 0.05 | 0.08 | 0.08 |
| CGM 30 min before | -0.02 |  |  |  |  | -0.02 | 0.03 | -0.01 |
| CGM 35 min before | 0.10 |  |  |  |  | -0.01 | -0.04 | -0.01 |
| CGM 40 min before | 0.01 |  |  |  |  |  | 0.08 | 0.03 |
| CGM 45 min before | -0.23 |  |  |  |  | 0.00 | -0.01 | -0.02 |
| CGM 50 min before | 0.19 |  |  |  |  | 0.01 | 0.05 |  |
| CGM 55 min before | -0.02 |  |  |  |  |  | 0.02 |  |
| CGM 60 min before | 0.03 | 0.05 |  |  |  |  | 0.03 |  |
| SD CGM during |  |  |  |  | 0.02 | 0.10 | -0.05 |  |
| Min CGM during |  |  |  |  | -0.16 | -0.09 | 0.11 | 0.02 |
| Max CGM during |  |  |  |  | -0.23 | -0.12 | 0.15 |  |
| Mean CGM during |  |  |  |  | 0.25 | 0.14 | -0.19 | 0.10 |
| CoV CGM during |  |  |  |  | 10.64 | 0.78 | 0.05 |  |
| Aerobic | -0.05 |  |  |  |  |  | 0.17 |  |
| Interval | -0.14 |  |  |  |  |  | 0.18 |  |
| Sex (female) | -0.05 |  |  |  |  |  | -0.21 |  |
| White | 0.00 |  |  |  |  |  | -0.23 |  |
| Asian | 0.00 |  |  |  |  |  | 0.15 |  |
| Black or African American | 0.00 |  |  |  |  |  | -0.10 |  |
| American Indian or Alaska Native | 0.00 |  |  |  |  |  | 0.03 |  |
| Race Unknown | 0.00 |  |  |  |  |  | -0.16 |  |
| Multiracial | 0.00 |  |  |  |  |  | 0.00 |  |
| A1C | -0.18 |  |  |  |  |  | 0.00 |  |
| Height | -0.07 |  |  |  |  |  | 0.03 |  |
| Weight | 0.02 |  |  |  | 0.01 |  | -0.01 |  |
| BMI | 0.04 |  |  |  |  |  | 0.10 |  |
| Morning | -0.24 | -0.76 |  |  |  |  | -0.01 |  |
| Night | 0.00 |  |  |  |  |  | 0.18 |  |
| Constant | 8.01 | 2.63 | -25.60 |  | -1.03 | 1.62 | -7.57 | -9.75 |

* For this model we report feature importance computed with Shapley Value instead of model coefficients.

| **Table S5: Model coefficients for models built with only the CGM data.** | | | | | | | | | | | | | | | | | | | | |
| --- | --- | --- | --- | --- | --- | --- | --- | --- | --- | --- | --- | --- | --- | --- | --- | --- | --- | --- | --- | --- |
| [executable version of the model files are included in this link](https://drive.google.com/drive/folders/1xDoDOfHWsOqKLJvqB-28VfUoTRokmkRJ?usp=drive_link) | | | | |  | |  | |  | |  | |  | |  | |  |  |  |  |
|  | During | | | | | | Post | | | | | | | |  |  |  |  |  |  |
|  | hypo <=54 | hypo <=70 | hyper >= 200 | | hyper >= 250 | | hypo <=54 | | hypo <=70 | | hyper >= 200 | | hyper >= 250 | |  |  |  |  |  |  |
| Classifier | svm | glm | glm | | glm | | glm | | svm | | glm | | glm | |  |  |  |  |  |  |
| SD CGM before | 0.04 | 0.01 |  | |  | | -0.26 | |  | | 0.00 | |  | |  |  |  |  |  |  |
| Min CGM before | 0.02 | -0.01 |  | |  | | -0.04 | |  | | 0.00 | |  | |  |  |  |  |  |  |
| Max CGM before | -0.03 | -0.07 |  | |  | | 0.03 | |  | | 0.00 | |  | |  |  |  |  |  |  |
| Mean CGM before | -0.05 | -0.10 |  | |  | | -0.44 | |  | | -0.31 | |  | |  |  |  |  |  |  |
| CoV CGM before | 0.01 | 11.71 |  | |  | | 22.12 | |  | | 1.97 | |  | |  |  |  |  |  |  |
| CGM 5 min before | -0.07 | -0.22 | 0.23 | | 0.28 | | 0.15 | | 0.02 | | 0.00 | | -0.06 | |  |  |  |  |  |  |
| CGM 10 min before | 0.07 | 0.13 |  | | -0.16 | | 0.15 | | 0.03 | | 0.02 | |  | |  |  |  |  |  |  |
| CGM 15 min before | -0.01 | 0.13 | -0.10 | |  | | -0.08 | | -0.02 | | 0.00 | |  | |  |  |  |  |  |  |
| CGM 20 min before | 0.05 | -0.05 |  | |  | | -0.03 | | -0.02 | | -0.03 | |  | |  |  |  |  |  |  |
| CGM 25 min before | -0.04 | 0.00 |  | |  | | 0.14 | | 0.04 | | 0.11 | |  | |  |  |  |  |  |  |
| CGM 30 min before | 0.00 | -0.03 |  | |  | | 0.05 | | 0.00 | | 0.03 | |  | |  |  |  |  |  |  |
| CGM 35 min before | 0.01 | 0.15 |  | |  | | -0.03 | | -0.02 | | -0.06 | |  | |  |  |  |  |  |  |
| CGM 40 min before | 0.03 | -0.02 |  | |  | | 0.02 | | 0.01 | | 0.11 | |  | |  |  |  |  |  |  |
| CGM 45 min before | -0.02 | -0.14 |  | |  | | 0.08 | | 0.00 | | -0.02 | |  | |  |  |  |  |  |  |
| CGM 50 min before | 0.01 | 0.11 |  | |  | | 0.12 | |  | | 0.05 | |  | |  |  |  |  |  |  |
| CGM 55 min before | -0.02 | 0.00 |  | |  | | -0.04 | |  | | 0.02 | |  | |  |  |  |  |  |  |
| CGM 60 min before | 0.04 | 0.05 |  | |  | | 0.05 | |  | | 0.02 | |  | |  |  |  |  |  |  |
| SD CGM during |  |  |  | |  | | 0.23 | | 0.10 | | -0.34 | |  | |  |  |  |  |  |  |
| Min CGM during |  |  |  | |  | | -0.09 | | -0.08 | | 0.10 | |  | |  |  |  |  |  |  |
| Max CGM during |  |  |  | |  | | -0.32 | | -0.11 | | 0.26 | | 0.02 | |  |  |  |  |  |  |
| Mean CGM during |  |  |  | |  | | 0.25 | | 0.12 | | -0.25 | | 0.08 | |  |  |  |  |  |  |
| CoV CGM during |  |  |  | |  | | 9.47 | | 0.09 | | 23.31 | |  | |  |  |  |  |  |  |
| Constant | -0.10 | 3.19 | -25.60 | | -30.75 | | -1.77 | | 1.95 | | -10.74 | | -10.55 | |  |  |  |  |  |  |

**Table S6: We compared our model performance to 6 models previously reported in the literature. Abbreviations in the table: Sen=Sensitivity, Spe=Specificity, Bal Acc=Balanced Accuracy. Different studies focused on different prediction targets, we used the model for the prediction target that is the most similar for the comparison. To enable easier comparison for sensitivity and specificity, we threshold our model prediction to closely match sensitivity reported in the corresponding prior study (labeled as “matching sensitivity”). We also report sensitivity and specificity for our model obtained by maximizing the J-index. Compared to the two in-clinic studies, our models have lower specificity when matching the sensitivity. This is likely due to the in-clinic studies has more frequent hypoglycemic events (Reddy et al reported hypoglycemic event rate 36%). Compared to other studies based on real-world data, our model performs similarly or better.**

| Comparison | Study | Target | Setting | AUROC | Sen | Spe | Bal Acc |
| --- | --- | --- | --- | --- | --- | --- | --- |
| 1 | Tyler et al^1^ | Post-Exercise (45 min) blood glucose<70 | In-clinic | NA | 0.71 | 0.94 | 0.83 |
|  | Our Study (matching sensitivity) | Post-Exercise (1 hr) blood glucose≤70 | Real-world | 0.89 | 0.73 | 0.89 | 0.81 |
|  | Our Study (J-Index) | Post-Exercise (1 hr) blood glucose≤70 | Real-world | 0.89 | 0.79 | 0.83 | 0.81 |
| 2 | Reddy et al^2^ | During Exercise blood glucose<70 | In-clinic | 0.97 | 0.98 | 0.97 | 0.97 |
|  | Our Study (matching sensitivity) | During Exercise blood glucose≤70 | Real-world | 0.91 | 1 | 0.38 | 0.69 |
|  | Our Study (J-Index) | During Exercise blood glucose≤70 | Real-world | 0.91 | 0.77 | 0.91 | 0.84 |
| 3 | Mosquera-Lopez et al^3^ | Post-Exercise Start (1 hr) blood glucose<70 | Real-world | 0.86 | 0.93 | 0.58 | 0.76 |
|  | Our Study (matching sensitivity) | During Exercise blood glucose≤70 | Real-world | 0.91 | 0.94 | 0.66 | 0.8 |
|  | Our Study (J-Index) | During Exercise blood glucose≤70 | Real-world | 0.91 | 0.77 | 0.91 | 0.84 |
| 4 | Bergford et al 2023^4^ | During Exercise blood glucose<70 | Real-world | 0.83 | 0.7 | 0.84 | 0.77 |
|  | Our Study (matching sensitivity) | During Exercise blood glucose≤70 | Real-world | 0.91 | 0.72 | 0.95 | 0.83 |
|  | Our Study (J-Index) | During Exercise blood glucose≤70 | Real-world | 0.91 | 0.77 | 0.91 | 0.84 |
| 5 | Bergford et al 2024^5^ | Post-Exercise Start (2 hr) blood glucose<70; Adolescent | Real-world | 0.79 | 0.74 | 0.74 | 0.74 |
|  | Our Study (matching sensitivity) | During Exercise blood glucose≤70; Adult | Real-world | 0.91 | 0.78 | 0.91 | 0.85 |
|  | Our Study (J-Index) | During Exercise blood glucose≤70; Adult | Real-world | 0.91 | 0.77 | 0.91 | 0.84 |
| 6 | Bergford et al 2024^5^ | Post-Exercise End (2 hr) blood glucose<70; Adolescent | Real-world | 0.84 | 0.9 | 0.68 | 0.79 |
|  | Our Study (matching sensitivity) | Post-Exercise (1 hr) blood glucose≤70; Adult | Real-world | 0.89 | 0.9 | 0.69 | 0.8 |
|  | Our Study (J-Index) | Post-Exercise (1 hr) blood glucose≤70; Adult | Real-world | 0.89 | 0.79 | 0.83 | 0.81 |

| 1 | Tyler, N.S., Mosquera-Lopez, C., Young, G.M., El Youssef, J., Castle, J.R., and Jacobs, P.G. (2022). Quantifying the impact of physical activity on future glucose trends using machine learning. iScience *25*, 103888. https://doi.org/10.1016/j.isci.2022.103888. |
| --- | --- |
| 2 | Reddy, R., Resalat, N., Wilson, L.M., Castle, J.R., El Youssef, J., and Jacobs, P.G. (2019). Prediction of Hypoglycemia During Aerobic Exercise in Adults With Type 1 Diabetes. J Diabetes Sci Technol *13*, 919–927. https://doi.org/10.1177/1932296818823792. |
| 3 | Mosquera-Lopez, C., Ramsey, K.L., Roquemen-Echeverri, V., and Jacobs, P.G. (2023). Modeling risk of hypoglycemia during and following physical activity in people with type 1 diabetes using explainable mixed-effects machine learning. Comput Biol Med *155*, 106670. https://doi.org/10.1016/j.compbiomed.2023.106670. |
| 4 | Bergford, S., Riddell, M.C., Jacobs, P.G., Li, Z., Gal, R.L., Clements, M.A., Doyle, F.J., Martin, C.K., Patton, S.R., Castle, J.R., et al. (2023). The Type 1 Diabetes and EXercise Initiative: Predicting Hypoglycemia Risk During Exercise for Participants with Type 1 Diabetes Using Repeated Measures Random Forest. Diabetes Technol Ther *25*, 602–611. https://doi.org/10.1089/dia.2023.0140. |
| 5 | Bergford, S., Riddell, M.C., Gal, R.L., Patton, S.R., Clements, M.A., Sherr, J.L., and Calhoun, P. (2024). Predicting Hypoglycemia and Hyperglycemia Risk During and After Activity for Adolescents with Type 1 Diabetes. Diabetes Technol Ther. https://doi.org/10.1089/dia.2024.0061. |

**Table S7. Calibration performance before and after applying three different recalibration methods. Performances are excellent. The ungrouped estimates represent nested cross validation Brier score estimates when exercise episodes are randomly assigned into different cross validation folds. This is the primary analysis reported in the main text of the paper and Table 4. The grouped estimates represent nested cross validation Brier score estimates when exercise episodes from the same participant are grouped together and randomly assigned into different cross validation folds**

| **Estimates** | **Event Type** | | | **Brier Score** | | | |
| --- | --- | --- | --- | --- | --- | --- | --- |
|  |  |  |  | **No Calibration** | **Isotonic Recalibration** | **Platt Recalibration** | **Spline Recalibration** |
| **Ungrouped** | **During** | **Hypo** | **≤54** | 0.017+/-(0.008) | 0.006+/-(0.003) | 0.006+/-(0.002) | 0.007+/-(0.003) |
|  |  |  | **≤70** | 0.035+/-(0.009) | 0.034+/-(0.010) | 0.031+/-(0.010) | 0.031+/-(0.010) |
|  |  | **Hyper** | **≥200** | 0.040+/-(0.034) | 0.029+/-(0.006) | 0.045+/-(0.040) | 0.028+/-(0.006) |
|  |  |  | **≥250** | 0.010+/-(0.000) | 0.011+/-(0.004) | 0.010+/-(0.002) | 0.015+/-(0.005) |
|  | **Post** | **Hypo** | **≤54** | 0.033+/-(0.005) | 0.033+/-(0.011) | 0.033+/-(0.012) | 0.034+/-(0.011) |
|  |  |  | **≤70** | 0.077+/-(0.001) | 0.072+/-(0.014) | 0.072+/-(0.014) | 0.078+/-(0.015) |
|  |  | **Hyper** | **≥200** | 0.075+/-(0.001) | 0.080+/-(0.009) | 0.079+/-(0.011) | 0.083+/-(0.008) |
|  |  |  | **≥250** | 0.028+/-(0.001) | 0.023+/-(0.006) | 0.022+/-(0.007) | 0.027+/-(0.009) |
| **Grouped** | **During** | **Hypo** | **≤54** | 0.020+/-(0.007) | 0.014+/-(0.003) | 0.015+/-(0.003) | 0.014+/-(0.003) |
|  |  |  | **≤70** | 0.038+/-(0.009) | 0.033+/-(0.002) | 0.039+/-(0.008) | 0.036+/-(0.004) |
|  |  | **Hyper** | **≥200** | 0.026+/-(0.003) | 0.023+/-(0.006) | 0.023+/-(0.007) | 0.029+/-(0.008) |
|  |  |  | **≥250** | 0.009+/-(0.001) | 0.013+/-(0.005) | 0.012+/-(0.003) | 0.016+/-(0.005) |
|  | **Post** | **Hypo** | **≤54** | 0.035+/-(0.005) | 0.033+/-(0.009) | 0.032+/-(0.010) | 0.033+/-(0.008) |
|  |  |  | **≤70** | 0.075+/-(0.001) | 0.068+/-(0.003) | 0.066+/-(0.002) | 0.069+/-(0.002) |
|  |  | **Hyper** | **≥200** | 0.077+/-(0.002) | 0.069+/-(0.007) | 0.066+/-(0.005) | 0.070+/-(0.005) |
|  |  |  | **≥250** | 0.027+/-(0.002) | 0.027+/-(0.006) | 0.026+/-(0.005) | 0.027+/-(0.006) |

**Table S8: We assessed the predictivity of the one CGM value immediately preceding exercise for predicting during exercise glycemic events and the one CGM value immediately preceding post exercise for predicting post exercise glycemic events. We refer to these models as “1 CGM” models. These models are analogous to using the single blood glucose measurement via a finger prick test (less resource intensive in terms of not requiring a CGM device, but perhaps more user burden since the measurement is not taken automatically and the glucose value need to be manually inputted into the model for prediction). Compared to the CGM only models with multiple CGM values as input, the 1 CGM models performs similarly for predicting during exercise glycemic events, except for hypo =<70, where the 1 CGM model performed worse. For predicting post exercise glycemic events, the 1 CGM models performed worse, except for predicting hyper >=250, where the 1 CGM and multiple CGM models performed similarly.**

|  | | **1 CGM** | **Multiple CGM** |
| --- | --- | --- | --- |
| during | hypo <=54 | 0.910+/-(0.007) | 0.906+/-(0.017) |
|  | hypo <=70 | 0.878+/-(0.001) | 0.912+/-(0.007) |
|  | hyper >= 200 | 0.980+/-(0.000) | 0.983+/-(0.006) |
|  | hyper >= 250 | 0.991+/-(0.000) | 0.989+/-(0.005) |
| post | hypo <=54 | 0.864+/-(0.001) | 0.901+/-(0.011) |
|  | hypo <=70 | 0.863+/-(0.000) | 0.894+/-(0.003) |
|  | hyper >= 200 | 0.885+/-(0.000) | 0.898+/-(0.002) |
|  | hyper >= 250 | 0.919+/-(0.000) | 0.919+/-(0.007) |
